# Stephanine Protects Against Osteoporosis by Suppressing Osteoclastogenesis via Inhibition of the RANKL—RANK Interaction

**DOI:** 10.1111/jcmm.70256

**Published:** 2024-12-05

**Authors:** Titi Liu, Jin Li, Meiyan Duan, Ya Wang, Zhe Jiang, Chunxia Gan, Zemin Xiang, Jun Sheng, Xuanjun Wang, Huanhuan Xu

**Affiliations:** ^1^ College of Science Yunnan Agricultural University Kunming China; ^2^ Key Laboratory of Pu‐Er Tea Science, Ministry of Education Yunnan Agricultural University Kunming China; ^3^ College of Food Science and Technology Yunnan Agricultural University Kunming China

**Keywords:** osteoclastogenesis, osteoporosis, RANK signalling, RANKL–RANK interaction, Stephanine

## Abstract

The interaction between the receptor activator of nuclear factor‐κB ligand (RANKL) and its receptor RANK is known to regulate osteoclastogenesis in bone remodelling and has become an important therapeutic target for the treatment of osteoporosis. Stephanine (SA), an isoquinoline aporphine‐type alkaloid isolated from *Stephania* plants, possesses excellent anti‐inflammatory effects and can be used for rheumatoid arthritis treatment. However, its specific role in osteoclastogenesis and osteoporosis remains unknown. In this study, we investigated the influence of SA on osteoclastogenesis in RANKL‐stimulated RAW 264.7 cells and osteoporosis in an ovariectomised (OVX) mouse model and elucidated the underlying molecular mechanism. In vitro, SA can bind to RANK and RANKL with the *K*_D_ values of 3.7 and 76.47 μM, respectively, and disrupt the RANKL–RANK interaction, which inhibits RANKL‐stimulated RANK–tumour necrosis factor receptor associated factor 6 (TRAF6) binding and RANK signalling pathways activation, downregulates the expression of key osteoclastogenesis‐related regulatory factors in osteoclast precursors, ultimately suppresses osteoclast differentiation and activation. In vivo, SA significantly ameliorated bone loss through inhibiting osteoclastogenesis in OVX mice because of the decreased number of osteoclasts and the increased trabecular bone area. SA markedly inhibited the serum levels of tartrate‐resistant acid phosphatase 5b (TRACP‐5b), c‐telopeptide of type I collagen (CTX‐I), and RANKL, whereas it increased that of osteoprotegerin (OPG) in OVX mice. Additionally, SA strikingly downregulated the OVX‐induced expression of osteoclast‐specific genes and proteins. Taken together, this study elucidated that SA can effectively protect against osteoporosis by suppressing osteoclastogenesis via inhibition of the RANKL–RANK interaction, which supports the potential application of SA as a natural therapeutic agent for osteoporosis.

## 1 Introduction

In life, bone is a dynamic organ that is continually remodelled, which maintains bone microstructure and rigidity [1, 2]. Bone remodelling is delicately controlled by osteoblast‐mediated bone formation and osteoclast‐mediated bone resorption [3]. The imbalance between bone formation and bone resorption can directly result in numerous metabolic bone diseases such as rheumatoid arthritis, osteopetrosis, and osteoporosis, which seriously endanger human health [4, 5]. Osteoporosis is a highly prevalent bone disorder characterised by reduced bone density and weakened bone microarchitecture, which lead to increased susceptibility to fractures [6]. Hyperactivated and excessive osteoclasts are closely related to the occurrence and development of osteoporosis in postmenopausal women [7]. As such, inhibiting osteoclastogenesis is an effective strategy for preventing and treating postmenopausal osteoporosis. Long‐term clinical anti‐osteoporosis drugs, such as calcium, bisphosphonates, denosumab, and other specific inhibitors, can effectively ameliorate bone loss, but are accompanied by serious adverse side effects on patients [8, 9]. Considering the limitation above, there is an urgent need to develop more specific and safer natural compounds sourced from medicinal plants for osteoporosis prevention and treatment, as well as explore the potential mechanisms.

Osteoclasts are multinucleated giant cells participating in bone resorption, which can adhere to the bone matrix and then secrete acid and lytic enzymes to degrade mineralised matrices [10]. They are originated from haematopoietic precursor cells of monocyte/macrophage lineage stimulated by cytoregulators such as macrophage colony‐stimulating factor (M‐CSF) and receptor activator of nuclear factor (NF)‐κB ligand (RANKL) [11, 12]. M‐CSF induces proliferation and survival of osteoclast precursor cells through binding to its receptor c‐Fms [13]. Meanwhile, RANKL stimulates mononuclear cell fusion to form large multinucleated osteoclasts [14, 15]. The binding of RANKL to its receptor RANK activates various intracellular signalling pathways, including phosphatidylinositol 3‐kinase/protein kinase B (AKT), mitogen‐activated protein kinases (MAPKs), and NF‐κB, by recruiting c‐Src and tumour necrosis factor receptor associated factor 6 (TRAF6) [16, 17]. Ultimately, these signalling cascades synergistically activate the key transcription factors nuclear factor of activated T cells 1 (NFATc1) and c‐Fos, as well as their target genes, such as c‐Src, cathepsin K, tartrate‐resistant acid phosphatase (TRAP), matrix metallopeptidase‐9 (MMP‐9), and β3‐Integrin, thereby promoting osteoclast differentiation and activation [18, 19]. Theoretically, it is feasible to inhibit the RANKL*–*RANK interaction directly from the source to suppress osteoclastogenesis and protect against osteoporosis, which has been recognised as a key and promising therapeutic strategy [20, 21, 22].

Recently, natural compounds derived from traditional herbs and medicinal plants have attracted much attention because of their protective effects on bone health [23, 24, 25]. However, very few natural agents with anti‐osteoporotic effects through blocking the RANKL–RANK interaction have been found. To explore the small molecules that could be used as lead compounds for anti‐osteoporosis drugs, we screened a library of compounds from medicinal plants using a surface plasmon resonance (SPR)‐based “A‐B‐A" competition assay. Then, we found that the natural isoquinoline aporphine‐type alkaloid stephanine (SA) exhibits excellent in vitro anti‐osteoclastogenesis activity. Alkaloids are the key active pharmaceutical ingredients in the *Stephania* plants [26]. It has been established that SA possesses multiple biological properties, including anti‐inflammatory, anti‐plasmodial, anti‐cancer, analgesic, and immunomodulatory activities [27, 28, 29, 30]. However, its specific role in osteoclastogenesis and osteoporosis remains unknown. In the present study, we revealed that SA can bind to RANKL and its receptor RANK and disrupt the RANKL–RANK interaction and that, via inhibition of RANKL‐stimulated RANK–TRAF6 binding and RANK signalling pathways activation, it effectively suppresses RANKL‐induced osteoclastogenesis in vitro and protects against osteoporosis in vivo. These findings highlight the potential application of SA as a natural small‐molecule compound against osteoporosis.

## 2 Materials and Methods

### 2.1 Reagents

Stephanine (molecular formula: C_19_H_19_NO_3_) of high purity grade (≥ 98%, Appendix S1) was obtained from BioBioPha Co. Ltd., (Kunming, China). Foetal bovine serum (FBS) and dulbecco's modified eagle's medium (DMEM) were provided by Shanghai XP Biomed Ltd. (VivaCell, Shanghai, China) and Meilun Biotechnology Co. Ltd., (Dalian, China), respectively. The recombinant mouse RANKL protein used in the cell experiments was purchased from R&D Systems (Minneapolis, MN, USA). Dimethyl sulfoxide (DMSO), TRAP staining kit, and 3‐(4,5‐dimethylthiazol‐2‐yl)‐2,5‐diphenyltetrazolium bromide (MTT) were obtained from Sigma‐Aldrich (St. Louis, MO, USA). Rhodamine‐phalloidin and antifade mounting medium with DAPI were sourced from Suzhou Yuheng Biotechnology Co. Ltd., (Suzhou, China). Anti‐tartrate‐resistant acid phosphatase 5b (TRACP‐5b) and anti‐β‐tubulin antibodies were sourced from ABclonal Technology (Wuhan, China). Antibodies against c‐Src, cathepsin K, NFATc1, c‐Fos, TRAF6, and RANK were purchased from Santa Cruz Biotechnology (Santa Cruz, CA, USA). Specific antibodies against signalling cascade molecules were obtained from Cell Signalling Technology (Beverly, MA, USA). Mouse TRACP‐5b, osteocalcin (OCN), c‐telopeptide of type I collagen (CTX‐I), and procollagen I N‐terminal peptide (PINP) ELISA kits were sourced from Cusabio Technology (Houston, TX, USA). Mouse RANKL and osteoprotegerin (OPG) ELISA kits were obtained from Boster Biological Technology Co. Ltd., (Wuhan, China).

### 2.2 Cell Line and Cell Culture

RAW 264.7 monocytic cell line was obtained from the Shanghai Cell Center (Shanghai, China) and cultured in DMEM containing 10% FBS and 1% penicillin–streptomycin (P/S; Solarbio) in an incubator (BINDER; Tuttlingen, Germany) with 5% CO_2_ at 37°C.

### 2.3 TRAP Staining

RAW 264.7 cells (2 × 10^3^ cells/well) were seeded into 96‐well plates and incubated overnight. Next, the cells were exposed to DMEM containing 6% FBS at different concentrations of SA (0, 2.5 or 5 μM) for 20 min and then treated with RANKL (50 ng/mL) for 4 days. Subsequently, TRAP staining was performed to determine the number of mature osteoclasts per well. Mature osteoclasts were defined as TRAP‐positive multinucleated cells with more than three nuclei under a microscope.

### 2.4 Immunofluorescence Staining

RAW 264.7 cells (2 × 10^4^ cells/well) were seeded into glass coverslips in a 12‐well plate and incubated overnight. Next, the cells were exposed to DMEM containing 6% FBS at different concentrations of SA (0, 2.5 or 5 μM) for 20 min and then treated with RANKL (50 ng/mL) for 6 days. Subsequently, immunofluorescence staining with rhodamine‐phalloidin and DAPI was performed to detect the number of F‐actin rings per well.

### 2.5 Cell Viability Assay

RAW 264.7 cells (1.5 × 10^4^ cells/well) were seeded into 96‐well plates and incubated overnight. After incubation for 24, 48 or 72 h in the presence of SA (0, 1, 2.5 or 5 μM), MTT assay was performed to examine cell viability.

### 2.6 qRT‐PCR Analysis

RAW 264.7 cells (3 × 10^5^ cells/well) were seeded into 12‐well plates and incubated overnight. Next, the cells were exposed to DMEM containing 6% FBS at different concentrations of SA (0, 2.5 or 5 μM) for 20 min and then treated with RANKL (50 ng/mL) for 72 h. RNA was isolated from cells or tissues using TRIzol reagent (Invitrogen, Carlsbad, CA, USA), and 1 μg of total RNA template was reverse‐transcribed into single‐stranded cDNA using Evo M‐MLV RT Mix Kit with gDNA Clean for qPCR (Accurate Biology, Changsha, China). The primers were verified in our previous studies [31, 32]. qRT‐PCR was conducted with SYBR Green Premix Pro Taq HS qPCR Kit (Accurate Biology) on a LightCycler 480 II PCR system (Roche, Basel, Switzerland).

### 2.7 Western Blotting Analysis

After treatment, total proteins were extracted from cells or tissues using radioactive immunoprecipitation assay lysis buffer (Solarbio). Equal amounts of protein extract (30 μg) were separated using sodium dodecyl sulphate‐polyacrylamide gel electrophoresis (SDS‐PAGE) and transferred onto polyvinylidene fluoride (PVDF) membranes (Millipore; Merck KGaA, Darmstadt, Germany). The membranes were incubated with specific primary antibodies overnight at 4°C and then incubated with anti‐rabbit or anti‐mouse secondary antibodies. Protein bands were developed using a super‐sensitive ECL chemiluminescent substrate (Biosharp, Hefei, China).

### 2.8 SPR Analysis

A Biacore S200 system (GE Healthcare) was used to measure molecular interaction. The RANK (uniport ID: Q9Y6Q6) and RANKL (uniport ID: O14788) proteins (Sino Biological Inc., Beijing, China) were diluted in sodium acetate buffer (pH 5.0), and then immobilised on the flow cell‐2 and ‐4 of a CM5 Sensor Chip via amine coupling protocol to reach the levels of 12,817 and 17,435 response units, respectively. The running buffer was PBS‐P supplemented with 5% DMSO. Different dilutions of SA were injected with an association time of 90 s and a dissociation time of 90 s, respectively. The kinetic parameters were calculated with Biacore S200 Evaluation Software using the kinetic analysis method. In addition, a well‐established solution competition experiment as described previously was performed to detect the inhibitory effect of SA on the RANKL–RANK interaction [33, 34].

### 2.9 Co‐Immunoprecipitation Analysis

RAW 264.7 cells (2 × 10^6^ cells/well) were seeded into 6‐well plates and incubated overnight. Next, the cells were exposed to DMEM containing 6% FBS at different concentrations of SA (0 or 5 μM) for 3 h. After treatment with RANKL (50 ng/mL) for 20 min, the cells were lysed and centrifuged at 15,000 *g* for 15 min. Subsequently, the anti‐RANK antibody was added to the supernatant and incubated overnight with rotation. Protein A/G PLUS‐Agarose beads (Santa Cruz Biotechnology) were then added and incubated with rotation for 4 h. Finally, the proteins after centrifugation were subjected to Western blotting analysis.

### 2.10 Animal Studies

All animal studies were approved by the Yunnan Agricultural University's Animal Care and Use Committee (approval number: YNAU2020LLWYH005–1). Thirty female C57BL/6 mice aged 8 weeks (Cawens, Changzhou, China) were subjected to either a sham operation or bilateral ovariectomy and assigned randomly to three groups: Sham, ovariectomised (OVX) and OVX + SA. These mice were intraperitoneally injected with 2 mg/kg body weight of SA or vehicle (PBS supplemented with 2.5% DMSO, 5% Tween 80 and 5% PEG400) every 2 days. After 6 weeks, the blood samples were obtained for serum biochemistry. The left/right femurs, livers and kidneys were collected for measurement of the organ index or histological analysis. Additionally, the tibiae were resected for qRT‐PCR and Western blotting analysis.

### 2.11 Histological Analysis

Fresh liver, kidney and femur tissues were fixed in 10% neutral formaldehyde for 72 h. The femur tissues were then decalcified with an EDTA decalcifying agent for 4 weeks. Paraffin‐embedded tissues were cut into 4‐μm sections for haematoxylin and eosin (H&E) or TRAP staining.

### 2.12 Serum Biochemistry

Blood samples were centrifuged at 1500 *g* for 15 min. The sera were collected and then the serum levels of TRACP‐5b, CTX‐I, RANKL, OCN, PINP and OPG were measured using the corresponding ELISA kits.

### 2.13 Statistical Analysis

All data are presented as the mean ± standard error of the mean (SEM) of three independent experiments. Statistical significance was determined using the Student's *t*‐test and Tukey's *post hoc* test. Significance was considered to be *p* < 0.05.

## 3 Results

### 3.1 SA Inhibits RANKL‐Induced Osteoclast Differentiation and F‐Actin Ring Formation in RAW 264.7 Cells

**FIGURE 1:**
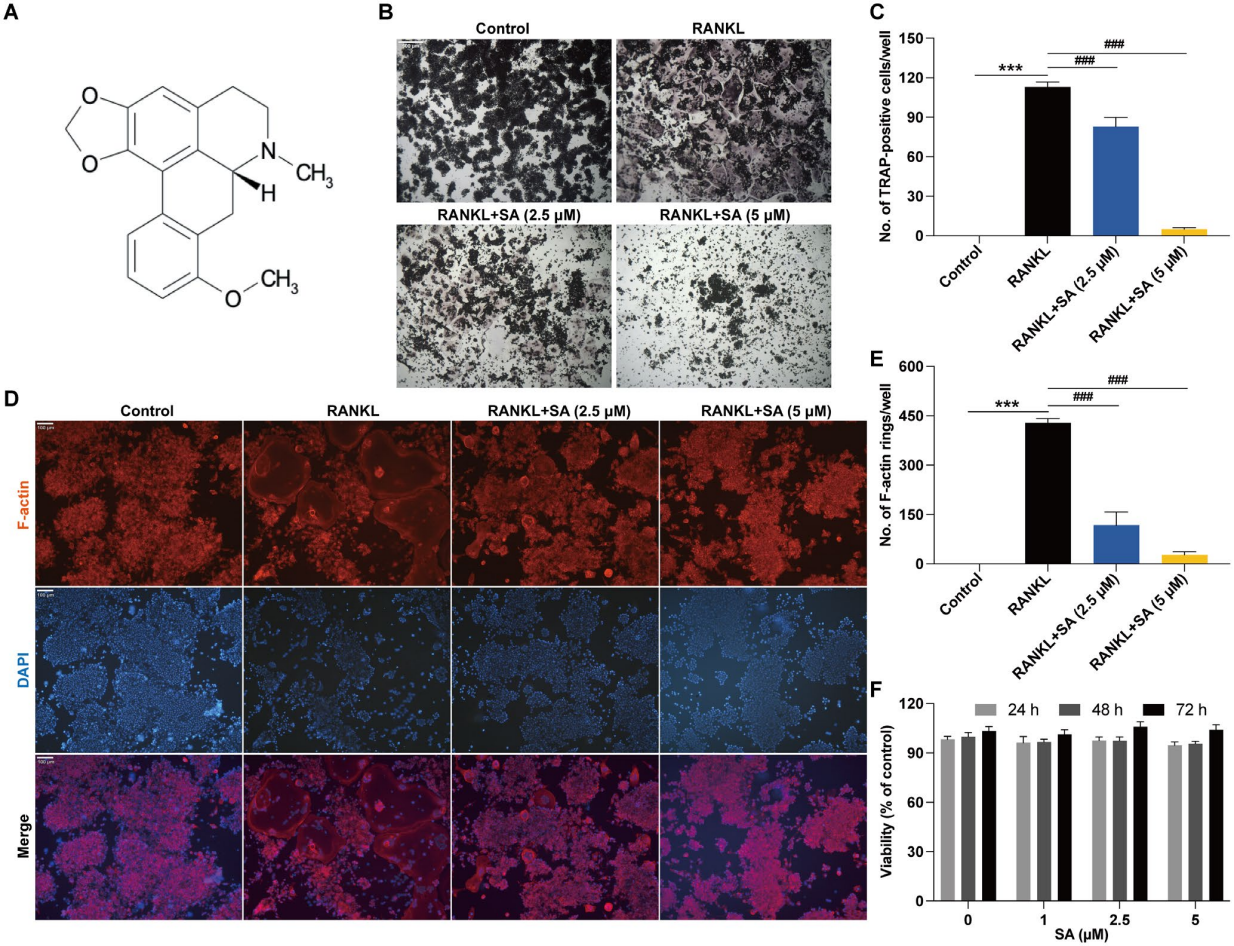
SA inhibits RANKL‐induced osteoclast differentiation and F‐Actin ring formation in RAW 264.7 cells. (A) The chemical structure of SA. (B) Representative images of TRAP staining showing that SA inhibited RANKL‐induced osteoclast differentiation in RAW 264.7 cells (original magnification ×40). (C) The TRAP‐positive multinucleated cells with more than three nuclei were counted as mature osteoclasts under a microscope. (D) Representative images of F‐Actin ring immunofluorescence staining showing that SA inhibited RANKL‐induced F‐Actin ring formation in RAW 264.7 cells (original magnification ×200). (E) The number of F‐Actin rings/well was calculated under a fluorescence microscope. (F) SA showed no cytotoxic effect on RAW 264.7 cells, as determined by MTT assay. Data are expressed as the mean ± SEM of three independent biological experiments. ***p < 0.001 versus the control group; ^###^ *p* < 0.001 versus the group only treated with RANKL.

The chemical structure of SA is shown in Figure 1A. To evaluate the influence of SA on osteoclastogenesis in vitro, we used the RANKL‐stimulated RAW 264.7 cells as the standard osteoclast differentiation model [35, 36]. As shown in Figure 1B, RANKL strikingly differentiated RAW 264.7 osteoclast precursors into mature osteoclasts, which was significantly hindered by SA in a concentration‐dependent manner (Figure 1C). The formation of F‐actin rings in mature osteoclasts is a characteristic marker of osteoclast‐mediated bone resorption [37]. Thus, we performed immunofluorescence staining to detect F‐actin ring formation. As shown in Figure 1D, SA notably and concentration‐dependently suppressed RANKL‐induced F‐actin ring formation (Figure 1E), suggesting that SA could negatively regulate the development and/or function of the mature osteoclasts. MTT assay revealed that SA was not cytotoxic on RAW 264.7 cells (Figure 1F), indicating that the inhibitory effects of SA on osteoclast differentiation and F‐actin ring formation were not mediated by its cytotoxicity.

### 3.2 SA Inhibits Osteoclastogenesis‐Related Marker Gene and Protein Expression

**FIGURE 2:**
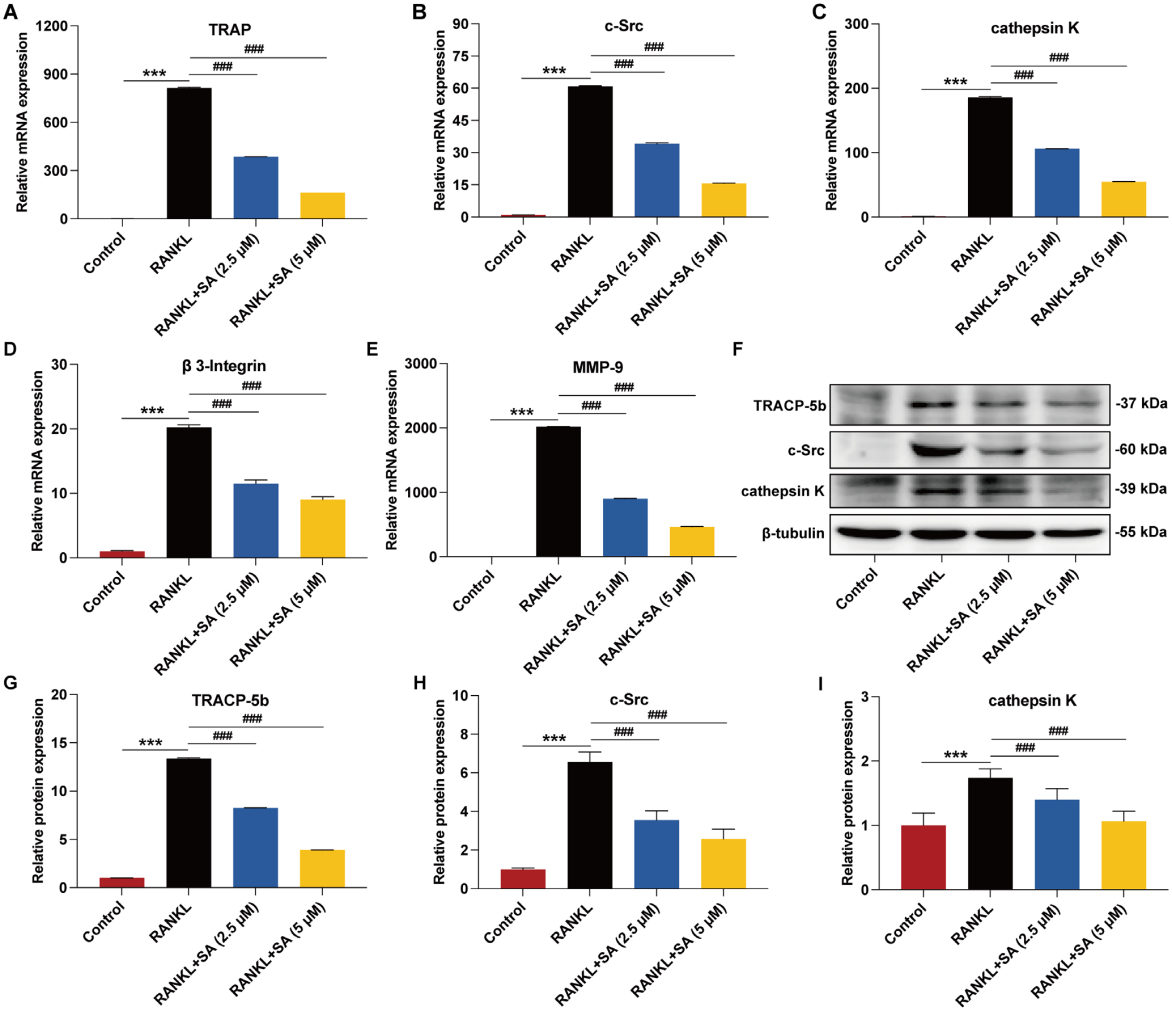
SA inhibits osteoclast‐specific gene and protein expression during osteoclastogenesis. (A–E) SA inhibited RANKL‐induced mRNA expression of TRAP (A), c‐Src (B), cathepsin K (C), β3‐Integrin (D) and MMP‐9 (E) in RAW 264.7 cells, as determined by qRT‐PCR analysis. (F) SA inhibited RANKL‐induced protein expression of TRACP‐5b, c‐Src and cathepsin K in RAW 264.7 cells, as determined by western blotting analysis. Representative immunoblots are displayed. (G–I) The relative protein expression corresponding to TRACP‐5b (G), c‐Src (H) and cathepsin K (I) was calculated using AlphaView software. Total RNA or proteins from RAW 264.7 cells pretreated with or without SA for 20 min, then stimulated with RANKL (50 ng/mL) for 72 h, were subjected to qRT‐PCR or western blotting analysis. Data are expressed as the mean ± SEM of three independent biological experiments. ***p < 0.001 versus the control group; ^###^ *p* < 0.001 versus the group only treated with RANKL.

The expression levels of related marker genes and proteins, such as TRAP, c‐Src, cathepsin K, β3‐Integrin and MMP‐9, contribute to osteoclastogenesis [38]. To elucidate the mechanism by which SA inhibits RANKL‐induced osteoclastogenesis, we determined the expression levels of these marker genes and proteins. qRT‐PCR revealed that SA strikingly and concentration‐dependently inhibited the RANKL‐stimulated mRNA expression levels of TRAP (Figure 2A), c‐Src (Figure 2B), cathepsin K (Figure 2C), β3‐Integrin (Figure 2D) and MMP‐9 (Figure 2E). Consistently, Western blotting revealed that the RANKL‐stimulated protein expression levels of TRACP‐5b, c‐Src and cathepsin K were notably and concentration‐dependently suppressed by SA (Figure 2F–I). These results demonstrated that SA inhibited osteoclast differentiation and activation via suppression of osteoclastogenesis‐related marker gene and protein expression.

### 3.3 SA Inhibits RANKL‐Stimulated Expression of Key Transcription Factors NFATc1 and c‐Fos

**FIGURE 3:**
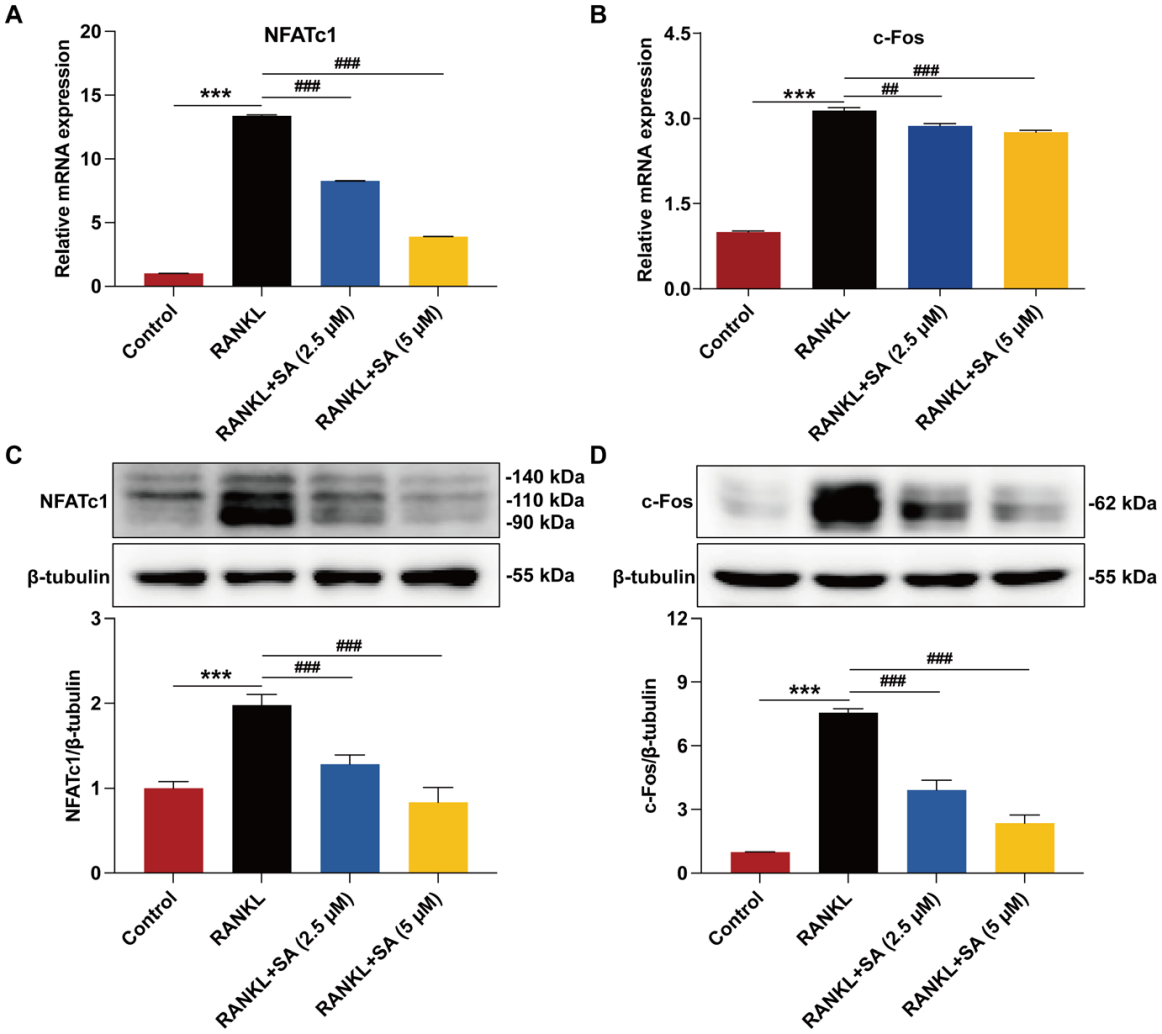
SA inhibits RANKL‐induced expression of key transcription factors NFATc1 and c‐Fos in RAW 264.7 cells. (A, B) SA inhibited RANKL‐induced mRNA expression of NFATc1 (A) and c‐Fos (B) in RAW 264.7 cells, as determined by qRT‐PCR analysis. (C and D) SA inhibited RANKL‐induced protein expression of NFATc1 (C) and c‐Fos (D) in RAW 264.7 cells, as determined by western blotting analysis. Representative immunoblots are displayed. The relative protein expression corresponding to NFATc1 and c‐Fos was calculated using AlphaView software. Treatment conditions were the same as described in Figure 2. Data are expressed as the mean ± SEM of three independent biological experiments. ***p < 0.001 versus the control group; ^##^ *p* < 0.01 and ^###^ *p* < 0.001 versus the group only treated with RANKL.

NFATc1 and c‐Fos, as the most important osteoclastogenic transcription factors, are typically induced by RANKL in osteoclast precursors and can induce osteoclast‐specific marker gene expression [39]. Since SA can inhibit osteoclastogenesis‐related marker gene expression, we next investigated whether SA inhibits the expression levels of NFATc1 and c‐Fos in RANKL‐stimulated RAW 264.7 cells as well. Not surprisingly, the RANKL‐stimulated mRNA expression levels of NFATc1 and c‐Fos in RAW 264.7 cells were significantly suppressed by SA as the concentration increased (Figure 3A,B). Consistently, the protein expression levels of NFATc1 and c‐Fos induced by RANKL were significantly suppressed by SA in a concentration‐dependent manner (Figure 3C,D).

### 3.4 SA Inhibits RANKL‐Induced RANK Signalling Pathways Activation

**FIGURE 4:**
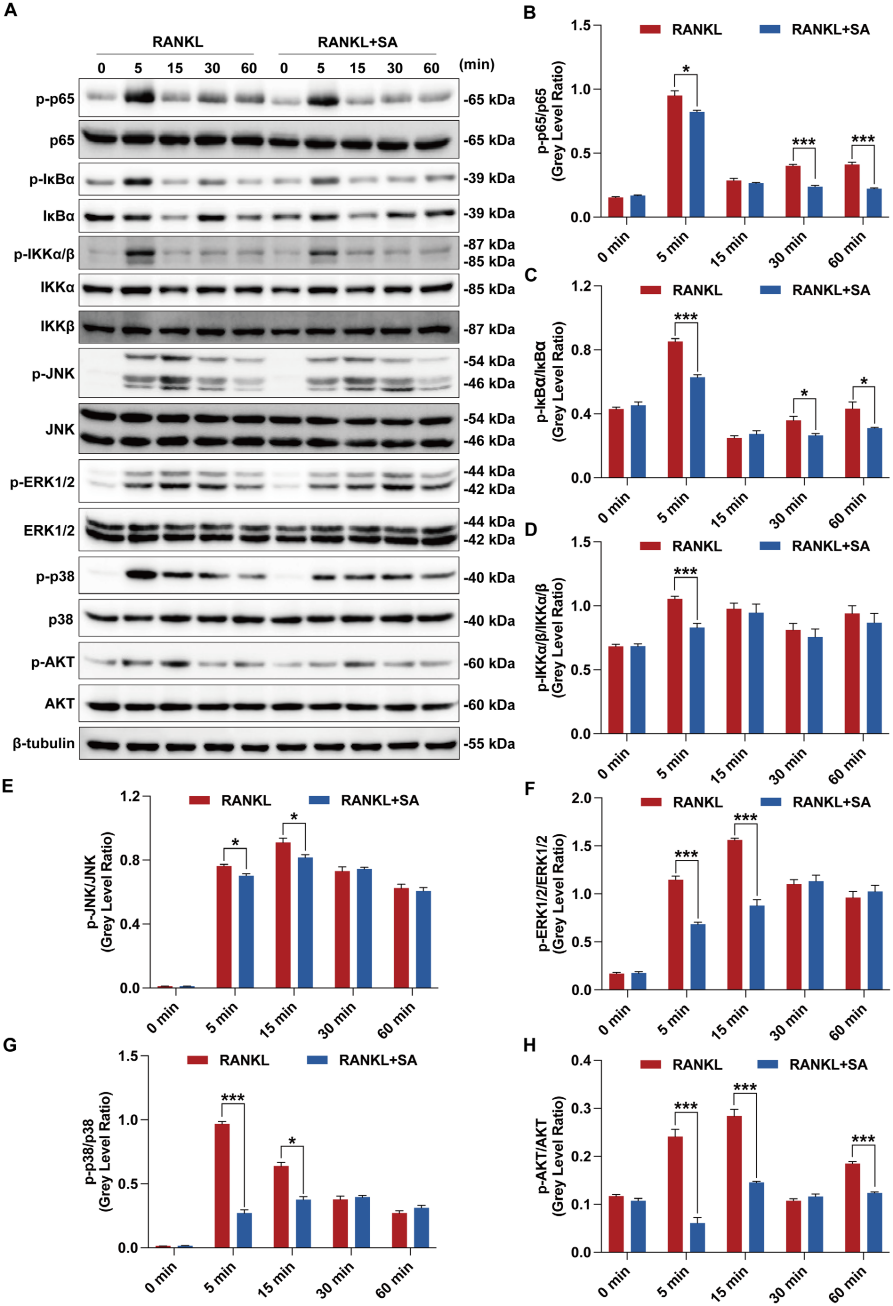
SA inhibits RANKL‐induced early canonical RANK signalling pathways in RAW 264.7 cells. (A) SA suppressed RANKL‐induced phosphorylation of p65, IκBα, IKKα/β, JNK, ERK1/2, p38 and AKT. RAW 264.7 cells were pretreated with or without SA (5 μM) for 3 h in serum‐free medium and then stimulated with RANKL (50 ng/mL) for the indicated treatment durations. Cell lysates were subjected to western blotting analysis using the indicated primary antibodies. Representative immunoblots are displayed. (B–H) The ratios of p65, IκBα, IKKα/β, JNK, ERK1/2, p38 and AKTrelative to total p65 (B), total IκBα (C), total IKKα/β (D), total JNK (E), total ERK1/2 (F), total p38 (G) and total AKT (H) were quantified using AlphaView software. Data are expressed as the mean ± SEM of three independent biological experiments. *p < 0.05 and ***p < 0.001 versus the group only treated with RANKL at the same time point.

RANKL can trigger the activation of NF‐κB, MAPK and AKT signalling pathways, culminating in the upregulation of NFATc1 and c‐Fos [1, 40]. Therefore, we further investigated whether SA inhibits RANKL‐mediated activation of RANK signalling pathways as well. Western blotting showed that RANKL obviously stimulated the phosphorylation of these signal transduction molecules (Figure 4A). As expected, SA significantly inhibited the phosphorylation of p65 (Figure 4B), IκBα (Figure 4C), IKKα/β (Figure 4D), JNK (Figure 4E), ERK1/2 (Figure 4F), p38 (Figure 4G) and AKT (Figure 4H) relative to total p65, total IκBα, total IKKα/β, total JNK, total ERK1/2, total p38 and total AKT in RANKL‐stimulated RAW 264.7 cells within 60 min. All these results demonstrated that SA could effectively inhibit RANKL‐induced activation of RANK signalling pathways.

### 3.5 SA Disrupts RANKL–RANK Interaction and Inhibits RANKL‐Stimulated RANK–TRAF6 Association

**FIGURE 5:**
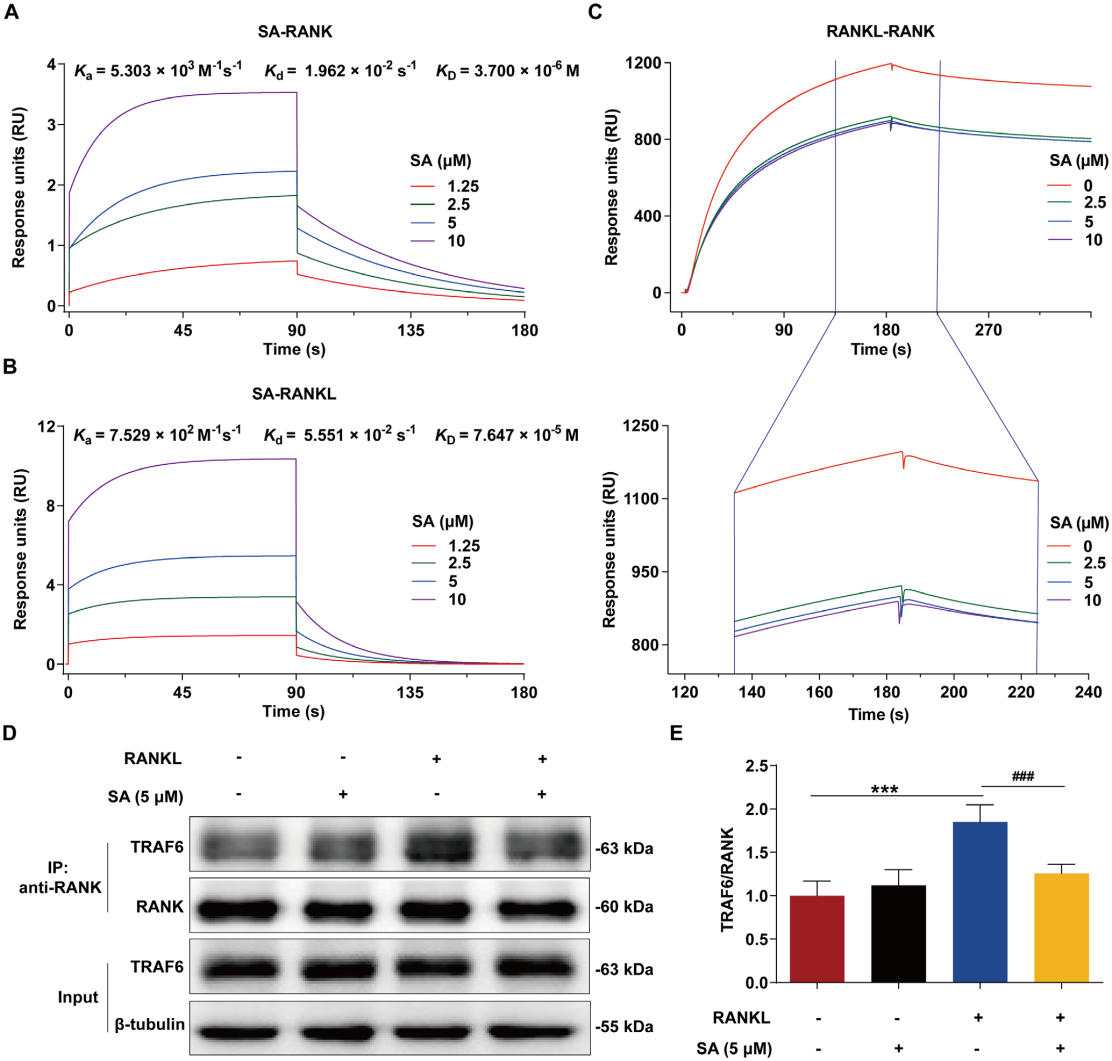
SA blocks RANKL–RANK interaction and inhibits RANKL‐induced RANK–TRAF6 association. (A and B) Direct interactions between SA and RANK (A) and RANKL (B) were determined by SPR kinetic analysis. (C) SA blocked the interaction between RANKL and RANK, as determined by SPR‐based “A‐B‐A" competition assay. The data represent one of three independent experiments with similar results. (D, E) SA suppressed RANKL‐stimulated interaction between RANK and TRAF6 in RAW 264.7 cells, as validated by co‐immunoprecipitation analysis. Representative immunoblots are displayed. The relative protein expression corresponding to TRAF6 was calculated using AlphaView software. Data are expressed as the mean ± SEM of three independent biological experiments. ***p < 0.001 versus the control group; ^###^ *p* < 0.001 versus the group only treated with RANKL.

Since the RANK signalling pathways are regulated by the binding of TRAF6 to RANK, which can be activated by RANKL [41, 42], we further investigated the possible role of SA in the interactions between RANK and RANKL and TRAF6 by SPR and co‐immunoprecipitation analysis. As shown in Figure 5A,B, SA is directly bound to RANK and RANKL with the *K*_D_ values of 3.7 and 76.47 μM, respectively. Additionally, kinetic data indicated that the *K*_a_ values were shown by RANK at 5.303 × 10^3^ M^−1^ s^−1^ and RANKL at 7.529 × 10^2^ M^−1^ s^−1^; the *K*_d_ values were showed by RANK at 1.962 × 10^−2^ s^−1^ and RANKL at 5.551 × 10^−2^ s^−1^. More importantly, the solution competition experiment revealed that SA could disrupt the RANKL–RANK interaction (Figure 5C). Given the inhibitory effect of SA on the RANKL–RANK interaction, we speculated that SA could inhibit the RANK–TRAF6 association in RANKL‐stimulated RAW 264.7 cells as well. As expected, RANKL promoted the interaction between RANK and TRAF6, which was markedly inhibited by SA (Figure 5D,E). Together, these data demonstrated that SA could disrupt RANKL–RANK interaction and inhibit RANKL‐stimulated RANK–TRAF6 association.

### 3.6 SA Prevents OVX‐Induced Bone Loss by Inhibiting Osteoclastogenesis

**FIGURE 6:**
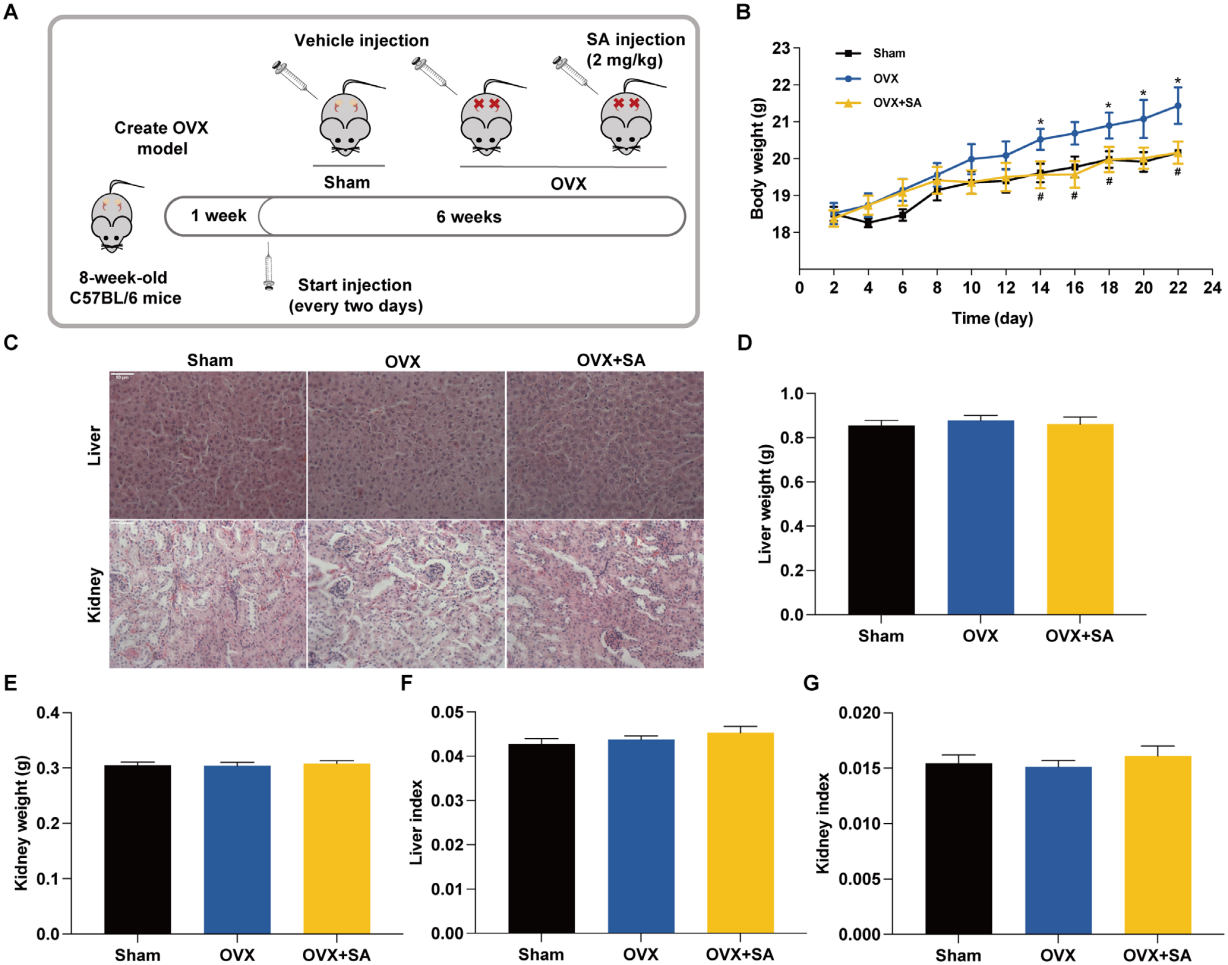
Treatment with SA does not have side effects on OVX mice. (A) Schematic representation of the in vivo experimental protocols. Body weight (B), liver and kidney histomorphology (C), liver weight (D), kidney weight (E), liver index (F) and kidney index (G) in each group were determined. Representative images are displayed (original magnification ×400). Data are expressed as the mean ± SEM (*n* = 10). *p < 0.05 versus the sham group; ^#^ *p* < 0.05 versus the OVX group.

**FIGURE 7:**
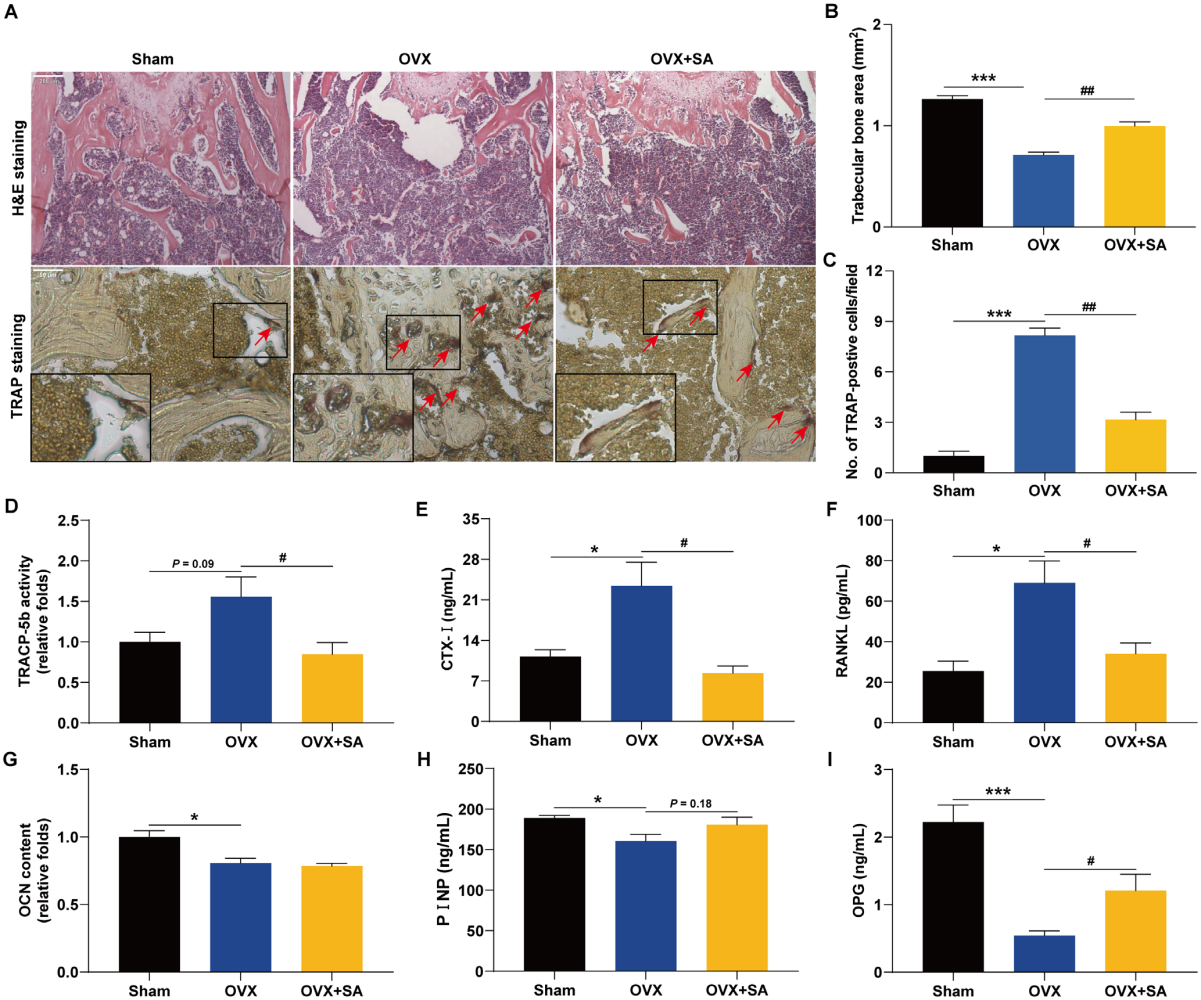
SA prevents OVX‐induced bone loss in mice by inhibiting osteoclastogenesis. (A) H&E staining (original magnification ×100) and TRAP staining (original magnification ×400) of the distal femur sections in each group. Representative images are displayed. (B) The trabecular bone area in the femur was quantified. (C) The number of TRAP‐positive cells/field was calculated. (D–I) The serum levels of TRACP‐5b (D), CTX‐I (E), RANKL (F), OCN (G), PINP (H) and OPG (I) were determined using the corresponding ELISA kits. Data are expressed as the mean ± SEM (*n* = 10). *p < 0.05 and ***p < 0.001 versus the sham group; ^#^ *p* < 0.05 and ^##^ *p* < 0.01 versus the OVX group.

**FIGURE 8:**
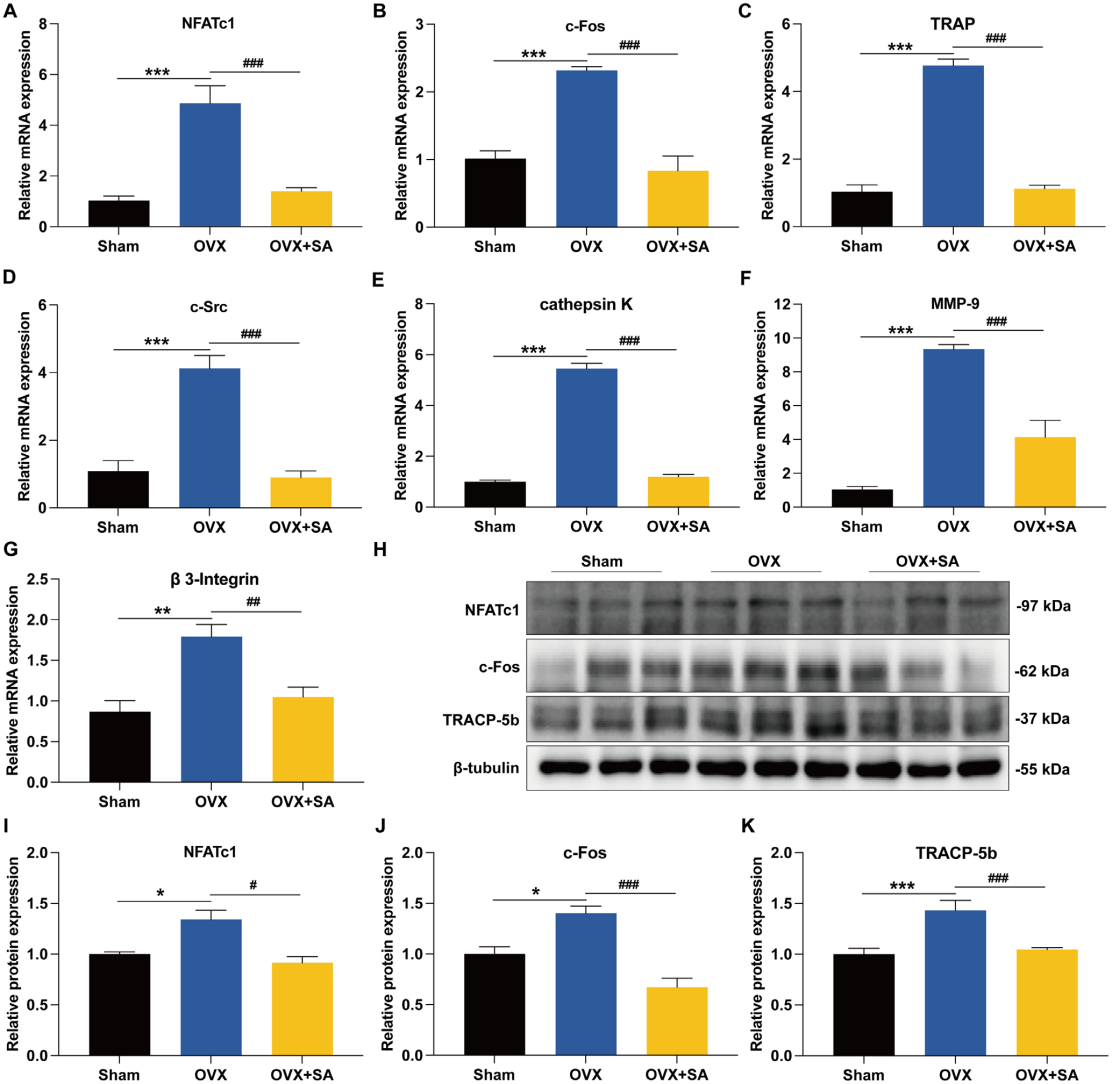
SA inhibits OVX‐induced expression of osteoclastogenesis‐related marker genes and proteins. (A–G) Treatment with SA inhibited OVX‐induced mRNA expression of NFATc1 (A), c‐Fos (B), TRAP (C), c‐Src (D), cathepsin K (E), MMP‐9 (F) and β3‐Integrin (G), as determined by qRT‐PCR analysis of the tibia tissues in each group. (H) Treatment with SA inhibited OVX‐induced protein expression of NFATc1, c‐Fos and TRACP‐5b, as determined by western blotting analysis of the tibia tissues in each group. Representative immunoblots are displayed. (I–K) The relative protein expression corresponding to NFATc1 (I), c‐Fos (J) and TRACP‐5b (K) was calculated using AlphaView software. Data are expressed as the mean ± SEM (*n* = 10). *p < 0.05, **p < 0.01 and ***p < 0.001 versus the sham group; ^#^ *p* < 0.05, ^##^ *p* < 0.01 ^###^ *p* < 0.001 versus the OVX group.

We further examined whether SA reduces bone loss using an OVX mouse model (Figure 6A), which can mimic oestrogen deficiency‐induced osteoporosis in women [43]. As shown in Figure 6B, treatment with SA significantly inhibited the OVX‐induced body weight gain. Additionally, SA did not exert toxic effects on the organs of the liver and kidney (Figure 6C–G). As shown in Figure 7A,B, SA strikingly increased the trabecular bone area in OVX mice. We further examined whether SA prevents OVX‐induced bone loss via inhibiting osteoclastogenesis. Interestingly, treatment with SA significantly inhibited osteoclast differentiation in OVX mice because of the decreased TRAP‐positive cells in the femur (Figure 7A,C). Moreover, OVX mice treated with SA displayed decreased serum TRACP‐5b (Figure 7D), CTX‐I (Figure 7E) and RANKL (Figure 7F), but OCN (Figure 7G) and PINP (Figure 7H) were not altered significantly compared with that in the OVX group, which suggested that SA ameliorated OVX‐induced bone loss through inhibition of osteoclastogenesis rather than by promoting osteogenesis. In addition, SA significantly increased the serum levels of OPG in OVX mice (Figure 7I). As shown in Figure 8A–G, treatment with SA significantly decreased the mRNA expression of NFATc1, c‐Fos, TRAP, c‐Src, cathepsin K, MMP‐9 and β3‐Integrin genes in OVX mice. Consistently, Western blotting revealed that SA significantly inhibited the OVX‐induced protein expression of NFATc1, c‐Fos and TRACP‐5b (Figure 8H–K). All these results demonstrated that SA could inhibit osteoclastogenesis in OVX mice and prevent oestrogen deficiency‐induced bone loss.

## 4 Discussion

**FIGURE 9:**
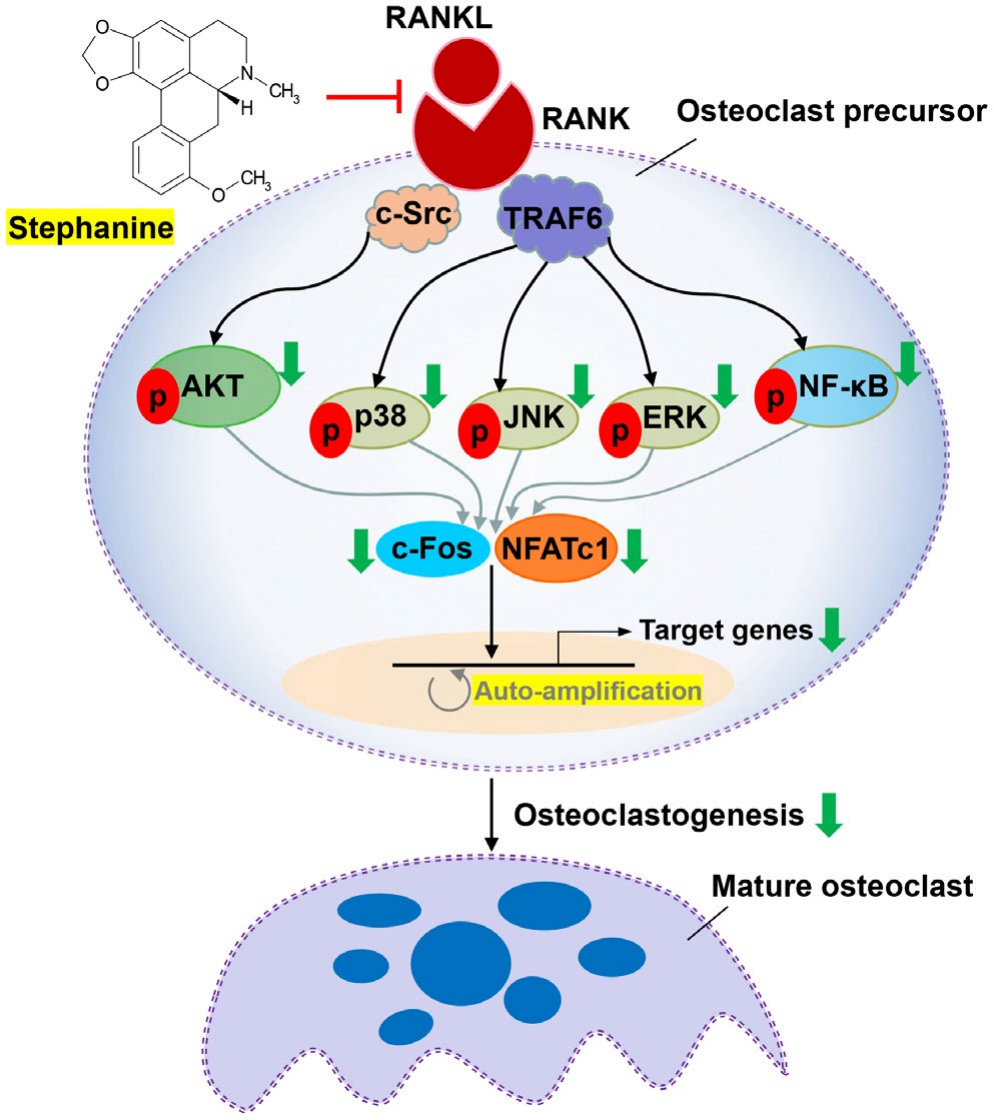
A schematic model of the molecular mechanisms underlying the inhibitory effect of SA on RANKL‐induced osteoclastogenesis.

Osteoporosis has become a serious global health issue that affects millions of individuals, especially postmenopausal women [44, 45]. The pathogenesis of osteoporosis is very slow, which is easily ignored by most people [46, 47]. It has been reported that osteoporosis affects more than 40% of postmenopausal women [48]. Osteoclast dysregulation or excessive osteoclastogenesis can directly result in osteoporosis [49]. The protein–protein interaction between RANKL and RANK is known to regulate osteoclastogenesis and is regarded as an important therapeutic target for the treatment of osteoporosis [50]. In this study, we found that SA, a natural small‐molecule compound, can bind directly to RANKL and its receptor RANK and disrupt the RANKL–RANK interaction and that, via inhibition of RANKL‐stimulated RANK–TRAF6 binding and RANK signalling pathways activation, it effectively suppresses osteoclastogenesis in RANKL‐stimulated RAW 264.7 cells (Figure 9) and prevents bone loss in an osteoporotic mouse model.

Natural medicinal plant‐derived products have become a reliable source for screening osteoclastogenesis inhibitors through targeted inhibition of RANKL–RANK interaction [51]. The genus *Stephania* is used as a traditional medicine against numerous diseases in China [27]. SA is an isoquinoline aporphine‐type alkaloid isolated from *Stephania* plants, which possesses excellent anti‐inflammatory effects and can be used for rheumatoid arthritis treatment [28, 29, 52]. Based on the above evidence and the results of SPR‐based target screening, we speculated that SA might suppress osteoclastogenesis via inhibition of the RANKL–RANK interaction and protect against oestrogen‐deficient osteoporosis. In our in vitro model, SA could significantly inhibit RANKL‐induced osteoclastogenesis. Mechanistically, SA directly interacted with the extracellular domains of RANK and RANKL, and the binding affinity between SA and RANK (*K*_D_ = 3.7 μM) was stronger than that with RANKL (*K*_D_ = 76.47 μM). More importantly, SA could be able to block the RANKL–RANK interaction, as demonstrated by the solution competition experiment. The results of the SPR studies suggested that SA could suppress osteoclastogenesis via inhibition of the RANKL–RANK interaction, although this needs further verification at the cellular level. Leucine‐rich repeat‐containing G‐protein‐coupled receptor 4 (LGR4), as a new RANKL receptor, can compete with RANK to bind RANKL and negatively regulate osteoclastogenesis [14]. The detailed interaction between SA and LGR4 requires further experimental studies.

RANKL stimulates osteoclast differentiation via binding to RANK, whose intracellular domain recruits c‐Src or TRAF6 to initiate signalling cascades such as AKT, MAPK and NF‐κB pathways [53, 54]. In the present study, SA could inhibit the RANK–TRAF6 binding and the phosphorylation of p65, IκBα, IKKα/β, JNK, ERK1/2, p38 and AKT, which is highly coherent with the RANKL–RANK interaction disrupted by SA. Based on these results, we speculated that SA might also inhibit the interaction between RANK and c‐Src, although this needs further investigation. The RANKL‐mediated RANK signalling pathways can trigger the induction of key osteoclastogenesis‐related transcription factors NFATc1 and c‐Fos [40, 55]. We observed that SA significantly inhibited the RANKL‐induced expression of NFATc1 and c‐Fos and their target genes. All these results indicated that SA could act as a natural small‐molecule osteoclastogenesis inhibitor via inhibition of the RANKL–RANK interaction. Collectively, this finding has the potential to broaden the availability of the therapeutic benefits of natural small‐molecule inhibitors of protein–protein interactions.

To mimic postmenopausal osteoporosis, an OVX‐induced bone loss model was established to verify the anti‐osteoporotic effect of SA. In this study, SA markedly inhibited the OVX‐induced body weight gain in mice [56]. In addition, SA did not exert toxic effects on the liver and kidney organs of OVX mice. H&E staining and TRAP staining of the distal femur sections indicated that SA effectively prevented OVX‐induced bone loss via inhibiting osteoclastogenesis because of the decreased number of osteoclasts and the increased trabecular bone area. More importantly, SA significantly improved blood indicators related to osteoclast activity. Consistently, qRT‐PCR and Western blotting analysis showed that treatment with SA effectively inhibited osteoclast activity in vivo via downregulation of the expression of osteoclastogenesis‐related marker genes and proteins.

Limitations within this study that indicate the need for future work. First, in vivo studies demonstrated that SA could ameliorate ovariectomy‐induced bone loss in mice. However, comparison of SA and anti‐osteoporosis drugs has not been clarified and will be addressed in future studies. Second, the pharmacokinetics of SA have yet to be elucidated. Third, the findings of this study highlight the potential application of SA as a natural small‐molecule compound against osteoporosis. However, further investigation is needed to evaluate the efficacy and safety of SA in preclinical studies or clinical trials. SA has many problems, such as poor bioavailability and poor water solubility. Thus, based on the chemical properties of SA, specific derivatives could be synthesised to search for more effective anti‐osteoporosis agents. Notably, cytoregulatory factors homologous to RANKL also play crucial roles in many other disorders such as rheumatoid arthritis [14], which are the subject of on‐going work in our laboratory.

## 5 Conclusion

Taken together, this study elucidated for the first time that SA can effectively protect against osteoporosis by suppressing osteoclastogenesis via inhibition of the RANKL–RANK interaction, which supports the potential application of SA as a natural therapeutic agent for osteoporosis.

## Author Contributions

**Titi Liu:** conceptualization (equal), project administration (equal). **Jin Li:** investigation (equal), writing – original draft (equal). **Meiyan Duan:** investigation (equal), software (equal). **Ya Wang:** formal analysis (equal), software (equal). **Zhe Jiang:** formal analysis (equal), methodology (equal). **Chunxia Gan:** investigation (equal), methodology (equal). **Zemin Xiang:** methodology (equal), resources (equal). **Jun Sheng:** funding acquisition (equal), resources (equal). **Xuanjun Wang:** project administration (equal), supervision (equal). **Huanhuan Xu:** funding acquisition (equal), supervision (equal), writing – review and editing (equal).

## Conflicts of Interest

The authors declare no conflicts of interest.

## Supporting information


Appendix S1.

## Data Availability

The data that support the findings of this study are available from the corresponding author upon reasonable request. More data that supports the findings of this study are available in Appendix S1 of this article.
