# Supplementary material for: Stephanine Protects Against Osteoporosis by Suppressing Osteoclastogenesis via Inhibition of the RANKL—RANK Interaction
Source: J Cell Mol Med. 2024 Dec 5;28(23):e70256. doi: 10.1111/jcmm.70256 (PMC11619157; doi:10.1111/jcmm.70256)
Supplement: Supplementary file 1 — Appendix S1. [file JCMM-28-e70256-s001.pdf]

西力生物
BBP BioBioPha

Natural Product Library for Drug Discovery

CERTIFICATE OF ANALYSIS

BBP No.: BBP00201

CAS No.: 517-63-5

Chemical Name: Stephanine

Molecular Formula: $C_{19}H_{19}NO_3$

Structure:

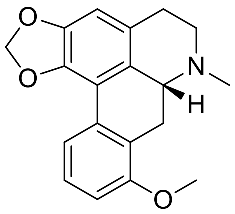

Purity: 98%

Appearance: Solid

Solvent: Acetone, methanol

Storage: Store in a dark place under the temperature of 0−4 °C

Intended Use: For laboratory use only

Reference: J. T. Blanchfield, et al., Phytochemistry, 2003, 63, 711-720

Warm Notice: When publishing, please cite as: ***chemical name*** was purchased from BioBioPha Co., Ltd. (Kunming, China)

Characterization Data Summary

Analytical Test	Results
Identification by ^{1}H-NMR	Consistent with the above structure
Purity tested by HPLC, ^{1}H-NMR	98%

Authorized Signature:

Date:

西力生物
BBP BioBioPha

Natural Product Library for Drug Discovery

PRODUCT QUALITY REPORT

Product Number: BBP00201

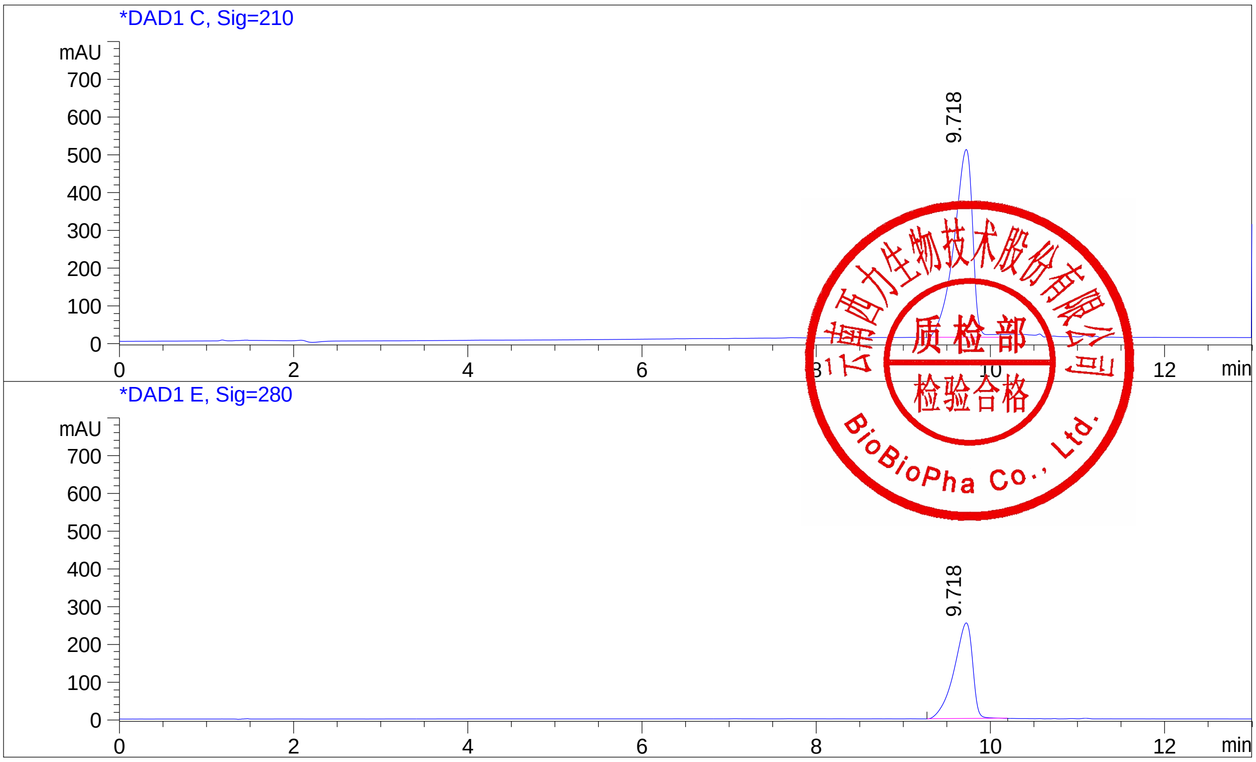

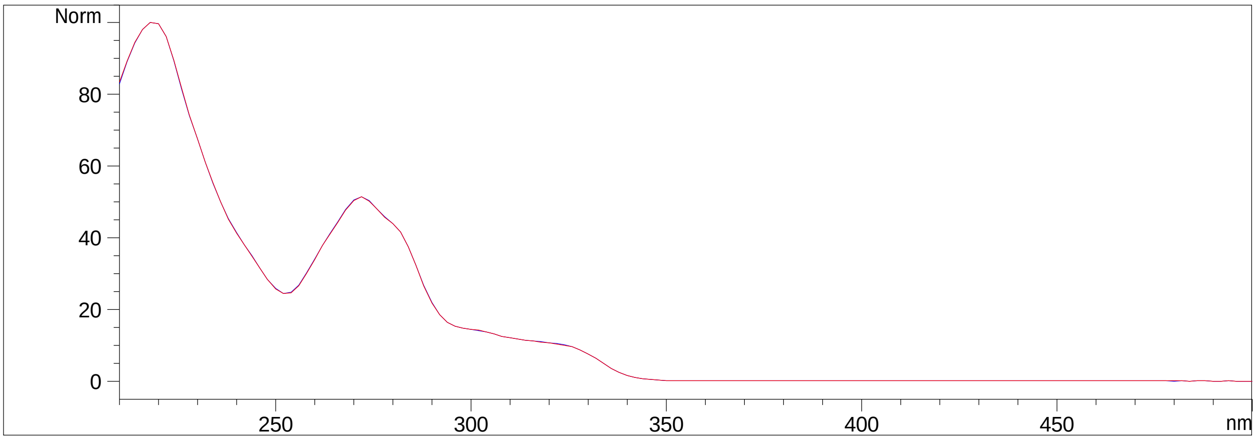

Agilent 1200 series HPLC system
Extend-C18 column (5 µm, 4.6 × 150 mm)
20%→100% MeOH in H_2O over 8.0 min followed by 100% MeOH to 13.0 min
1.0 ml/min, 20°C

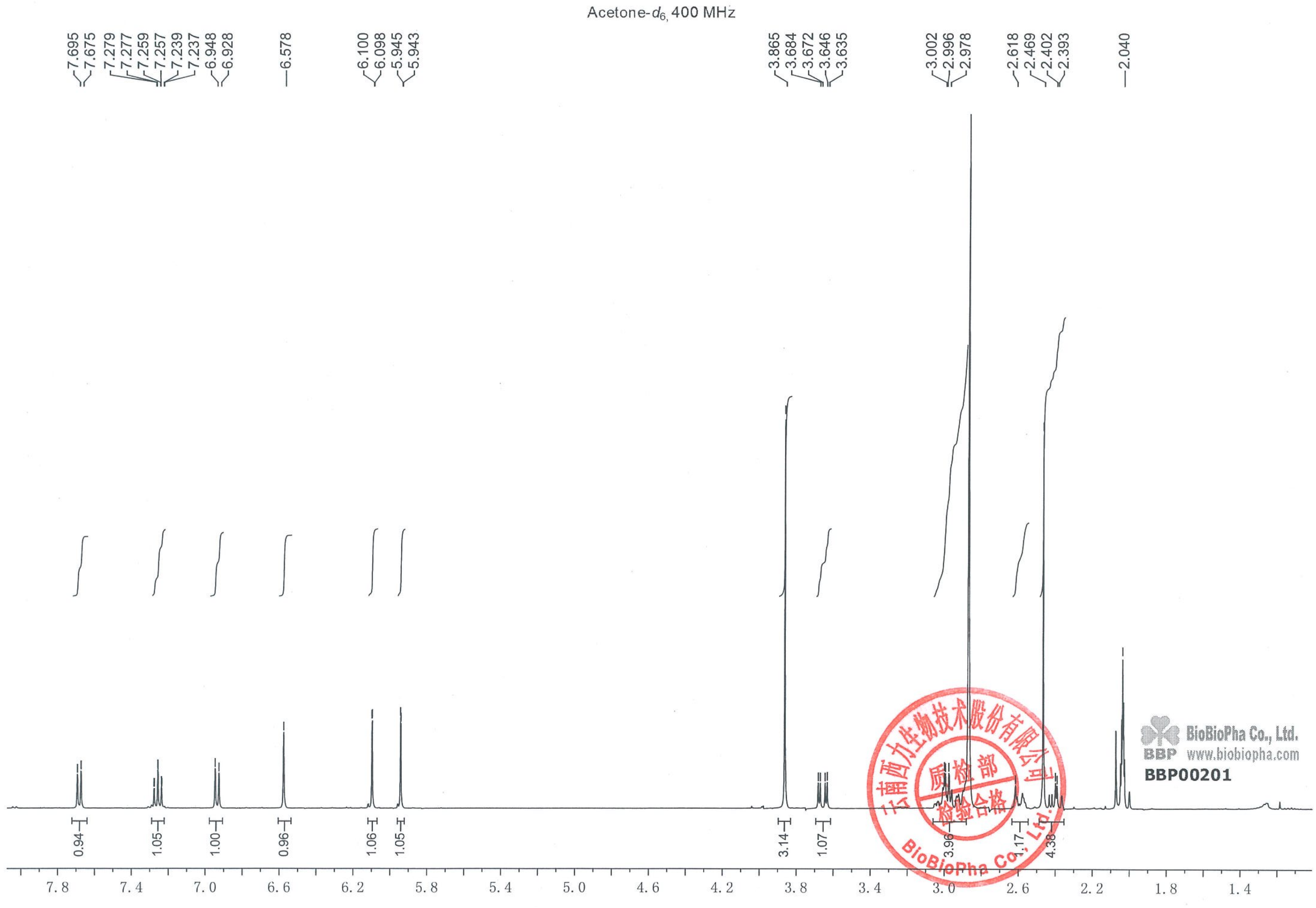
Acetone-d6, 400 MHz
7.695
7.675
7.279
7.277
7.259
7.257
7.239
7.237
6.948
6.928
6.578
6.100
6.098
5.945
5.943
3.865
3.684
3.672
3.646
3.635
3.002
2.996
2.978
2.618
2.469
2.402
2.393
2.040
0.94
1.05
1.00
0.96
1.06
1.05
3.14
1.07
3.96
1.17
4.38
7.8
7.4
7.0
6.6
6.2
5.8
5.4
5.0
4.6
4.2
3.8
3.4
3.0
2.6
2.2
1.8
1.4
BioBioPha Co., Ltd.
BBP www.biobiopha.com
BBP00201
云南西力生物技术股份有限公司
质检部
检验合格
BioBioPha Co., Ltd.
